# Multinomial analysis of behavior: statistical methods

**DOI:** 10.1007/s00265-017-2363-8

**Published:** 2017-08-25

**Authors:** Jeremy Koster, Richard McElreath

**Affiliations:** 1 Department of Anthropology, University of Cincinnati, Cincinnati, OH USA; 2 Max Planck Institute for Evolutionary Anthropology, Deutscher Platz 6, 04103 Leipzig, Germany; 3 Department of Anthropology, University of California, Davis, Davis, CA USA

**Keywords:** Generalized linear mixed models, Multinomial logistic regression, Scan sampling, Focal observations, RStan

## Abstract

Behavioral ecologists frequently use observational methods, such as instantaneous scan sampling, to record the behavior of animals at discrete moments in time. We develop and apply multilevel, multinomial logistic regression models for analyzing such data. These statistical methods correspond to the multinomial character of the response variable while also accounting for the repeated observations of individuals that characterize behavioral datasets. Correlated random effects potentially reveal individual-level trade-offs across behaviors, allowing for models that reveal the extent to which individuals who regularly engage in one behavior also exhibit relatively more or less of another behavior. Using an example dataset, we demonstrate the estimation of these models using Hamiltonian Monte Carlo algorithms, as implemented in the *RStan* package in the *R* statistical environment. The supplemental files include a coding script and data that demonstrate auxiliary functions to prepare the data, estimate the models, summarize the posterior samples, and generate figures that display model predictions. We discuss possible extensions to our approach, including models with random slopes to allow individual-level behavioral strategies to vary over time and the need for models that account for temporal autocorrelation. These models can potentially be applied to a broad class of statistical analyses by behavioral ecologists, focusing on other polytomous response variables, such as behavior, habitat choice, or emotional states.

**Electronic supplementary material:**

The online version of this article (doi:10.1007/s00265-017-2363-8) contains supplementary material, which is available to authorized users.

In both naturalistic and experimental contexts, observational methods are mainstays in the research designs of behavioral ecologists. The sampling methods for observational studies have been established for decades, and the canonical overview by Altmann (1974) has been cited thousands of times. Although continuous monitoring of focal individuals occasionally focuses on the timing of transitions between behavioral states (Martin and Bateson 2007), it is also common for behavioral ecologists to document the behavior of a sampled individual at a particular moment. The behaviors of multiple individuals may be documented simultaneously, as in instantaneous scan sampling, or the behavior of a focal individual may be recorded at regular, prespecified intervals. In both cases, the basic unit of analysis is the behavior that is exhibited by an individual at a given moment in time.

Ethograms, or coding schemes, vary considerably depending on the species being observed and the goals of the research. In rare cases, behavioral researchers may elect to record only two behaviors, perhaps contrasting a behavior of interest against a reference category that subsumes all other behaviors (e.g., foraging behavior versus all alternative behaviors). More commonly, however, behavioral researchers use ethograms in which they document *K* behaviors, where *K* is a quantity of behavioral categories that can vary considerably from study to study. An assortment of statistical approaches has been applied to these data, including regression analyses of aggregated proportions (or their principal components) and logistic regression of discrete, binarized behavioral categories (Isbell and Young 1993; Mainguy and Côté 2008; Singh et al. 2010; Willisch and Neuhaus 2010; Dantzer et al. 2012; Koster et al. 2013). Although informative, these statistical methods depart from the multinomial data structure that characterizes observational research, and they are incongruous with behavioral ecologists’ theoretical models of time budgets that center on trade-offs and opportunity costs. That is, time allocated to one behavior precludes time devoted to alternative, fitness-enhancing behaviors (Sharpe and Rosell 2003; Johnson and Bock 2004; Morrell 2004; Reaney 2007; Hamel and Côté 2008). Prevailing statistical methods are not well suited for elucidating these trade-offs and the ensuing individual-level correlations across behavioral categories.

The primary goal of this paper is to explain and promote a multilevel, multinomial logistic regression approach to the analysis of behavioral data. These statistical models correspond to the multinomial character of the response variable while also accounting for the repeated observations of individuals that typify behavioral datasets. Not only does our modeling approach account for the pseudoreplication stemming from repeated observations but also the correlated random effects potentially reveal individual-level covariation across behaviors. In other words, it is possible to comment on the extent to which individuals who regularly engage in one behavior also exhibit relatively more or less of another behavior.

The statistical methods that we promote are not an original development, but rather a repurposing of existing methods. To some extent, it is surprising that multinomial models for behavioral data are not more common given the ubiquitous use of multinomial logistic models for diverse ecological applications, such as vegetation analysis, community ecology, and parentage assignment (Augustin et al. 2001; Hadfield et al. 2006; Brienen et al. 2010; Hatala et al. 2011; Witter et al. 2012; Cristescu et al. 2015; Ackerly et al. 2015). Behavioral ecologists have likewise employed multinomial models for research topics such as habitat selection, food choices, and behavioral responses (Chancellor and Isbell 2008; May et al. 2008; Sagata and Lester 2009; Marshall et al. 2012). As conceptual understandings and software for multilevel (mixed-effects) models have advanced among behavioral ecologists, some researchers have recently begun to use the multilevel, multinomial model that we espouse in this paper to account for the repeated observations of individuals (Browning et al. 2012; Koster et al. 2015). To our knowledge, however, no previous study has reported the correlated random effects from these models, overlooking a potentially rich source of inferential insight into behavioral strategies and trade-offs.

In this paper, we provide an overview of our modeling approach, which we demonstrate via an analysis of ethnographic data collected using a variant of instantaneous scan sampling (Altmann 1974; Borgerhoff Mulder and Caro 1985). A secondary goal is to demonstrate the use of two relatively new *R* packages, *RStan* and *rethinking*, for model fitting and analysis (Stan Development Team 2016; McElreath 2015). It is possible to fit multinomial models in other packages, ranging from *R* packages such as *MCMCglmm* to general-purpose multilevel modeling software such as MLwiN (Hadfield 2010; Charlton et al. 2017). An advantage of *RStan* relative to other software is superior and faster estimation of models, courtesy of its Hamiltonian Monte Carlo algorithm (Monnahan et al. 2017). Whereas conventional MCMC chains potentially require millions of iterations to reach stationarity (e.g., Browning et al. 2012), our models require only a few thousand iterations to achieve an adequately mixed posterior distribution. In the supplemental files, we include the script and empirical data from our case study so that researchers can replicate our models and extend the modeling approach to new data.

To illustrate the models, we use a case study from observational data on the activities of adult and adolescent males in an indigenous Nicaraguan society. An additional substantive goal of this paper is to test hypotheses drawn from life history theory about age-related time allocation decisions among humans in subsistence-oriented societies. The interest in this question stems from the unique features of human life history traits, which are distinguished from primate life history traits by delayed onset of reproduction, comparatively brief inter-birth intervals, and an extended post-reproductive lifespan (Jones 2011). The adaptive origins of these traits plausibly lie in cooperative production strategies, as mates and post-reproductive individuals provide the resources needed to sustain women’s high reproduction (Hooper et al. 2015). For the maximization of group-level synergies and pooling of resources, individual members of kin groups should divide themselves among activities that capitalize on the comparative advantages that result from their respective combinations of physical abilities and acquired skills. Among indigenous Peruvians, younger individuals focus on low-strength, low-skill activities (e.g., domestic tasks) before transitioning to high-strength, high-skill activities in middle adulthood (e.g., hunting) and eventually as elders to activities that require advanced skills but fewer physical demands, such as agriculture and manufacture (Gurven and Kaplan 2006). The current analysis tests similar predictions in a different population of subsistence-level horticulturalists.

Owing to the focus on life history predictions, the analysis centers on age as a predictor of behavior. In addition to this individual-level variable, other covariates in the models include predictors that have parallels in behavioral ecology more generally, such as time-varying environmental predictors (e.g., rainfall), characteristics of the individuals’ residence groups (e.g., household size), and both continuous and categorical temporal controls (i.e., time of day and day of the week). We emphasize the importance of calculating and plotting model predictions to avoid the pitfalls that commonly arise from an overreliance on estimated coefficients for the interpretation of multinomial models. In addition to the basic models with random effects for individuals, we introduce models with random effects that reflect common sources of clustering in behavioral ecological datasets, specifically the clustering of individuals in social groups (e.g., Browning et al. 2012) and temporal units, such as the months or years in which behavior was recorded (e.g., Griesser and Nystrand 2009). These general considerations are accompanied by an emphasis on the most original aspect of this analysis, specifically the use of correlated random effects to understand individual-level trade-offs.

## The multilevel multinomial behavior model

For the basic multilevel multinomial behavior model (MMBM), we assume that ethograms are composed of *K* behavioral categories. By convention, we use positive integers to index these categories: 1, 2, 3, . . ., *K*. Following the categorical (generalized Bernoulli) distribution, the probability of observing each category *k* is defined as *π*_*k*_. One of these categories serves as the reference category around which other categories “pivot.” In other words, the model is composed of *K −* 1 equations that contrast the odds of exhibiting behavior *k* instead of the reference behavior.

It is common for ecologists to observe individuals on multiple occasions, which introduces pseudoreplication that necessitates statistical models to account for this higher-level clustering (Bolker et al. 2009). In the context of these multinomial models, the use of multilevel modeling allows the probabilities of exhibiting behavior *k* to vary across individuals. For each of the sub-equations, a random effect (varying intercept) is added that allows individuals to have greater or lesser odds of being observed in category *k* instead of the reference category. A noteworthy advantage of the multinomial approach is that we can estimate the correlations of these random effects across the *K* − 1 response categories, thus providing insights into the co-occurrence of different behaviors by individuals. In addition to these insights about co-occurrence, the correlations facilitate pooling of information across behavioral categories, reducing overfitting and improving estimates of parameters in the model.

For simplicity, imagine an ethogram that records only three possible behaviors (*k* = 1, 2, 3). The last category (*k* = 3) serves as the reference category. Assuming discrete observations at time *t*, the log-odds that individual *i* exhibits the remaining behaviors instead of the reference category is notated as

$$ {\displaystyle \begin{array}{c}\hfill \log \left(\frac{\pi_{1 it}}{\pi_{3it}}\right)={\beta}_{1 it}+{v}_{1i}\hfill \\ {}\hfill \log \left(\frac{\pi_{2 it}}{\pi_{3it}}\right)={\beta}_{2 it}+{v}_{2i}\hfill \\ {}\hfill \left[\begin{array}{c}\hfill {v}_{1i}\hfill \\ {}\hfill {v}_{2i}\hfill \end{array}\right]\sim \mathrm{Normal}\left(0,{\varOmega}_v\right):{\varOmega}_v=\left[\begin{array}{cc}\hfill {\sigma}_{v 1}^2\hfill & \hfill \hfill \\ {}\hfill {\sigma}_{v 1, 2}\hfill & \hfill {\sigma}_{v 2}^2\hfill \end{array}\right]\hfill \\ {}\hfill {\pi}_1+{\pi}_2+{\pi}_3=1\hfill \end{array}} $$

where *β*_1*it*_ and *β*_2*it*_ are the intercepts that contrast the first and second behaviors against the reference category, and *v*_1*i*_ and *v*_2*i*_ are the individual-level random effects, which are assumed to be multivariate normally distributed with zero means and a homogenous 2 × 2 variance-covariance matrix. For brevity, we present equations with only intercepts, but additional covariates (i.e., fixed effects) can be included to model the extent to which the individuals exhibit relatively more or less of the *k* behavior instead of the reference.

When an individual-level varying intercept is positive (*v*_k*i*_ > 0), it indicates that individual *i* has an above-average chance of exhibiting behavior *k* instead of the reference behavior. The inverse is true of varying intercepts that are negative. The above parameterization models the correlation of these random effects across the *K* − 1 response categories. In the above example, the correlation is derived per usual: *ρ*_1,2_ = *σ*_*v*1,2_/(*σ*_*v*1_ *σ*_*v*2_). The correlation is standardized to lie between − 1 and 1. When the correlation is positive, it indicates that individuals who do more of the first behavior also do more of the second behavior (relative to the reference category in both cases). A negative correlation implies that individuals who do relatively more of the first behavior do relatively less of the second behavior.

Whereas this model assumes an ethogram with only three behaviors, the model can be expanded to accommodate a greater number of behavioral categories. When *K* equals 10, for example, then there are nine sub-equations and a corresponding 9 × 9 variance-covariance matrix of the individual-level random effects. From the basic version of the MMBM in this example, behavioral ecologists therefore can generalize broadly to datasets in which individuals exhibit *K* possible behaviors at time *t*.

In addition to fixed effect covariates, this modeling approach can also accommodate additional random effects for hierarchical or cross-classified data structures. See Supplemental File 2 for simplified notation corresponding to models in this paper that include random effects for group membership and the temporal intervals in which observations occurred.

## Example dataset

We illustrate our modeling approach using observational data on the activities of 45 adolescent and adult males in a community of indigenous Nicaraguan horticulturalists (Koster et al. 2013; Koster and Leckie 2014). The data were collected during a 12-month study period in 2004–2005, with approximately 7 days per month devoted to data collection. Observations were scheduled during daylight hours and organized by household, as in other anthropological applications of scan sampling methods (Borgerhoff Mulder and Caro 1985). The initial observation was scheduled randomly between 5:30 and 6:00 a.m., and then subsequent observations were scheduled every 30 min, concluding no later than 6:00 p.m. During an observation, the lead author documented the activities of all household residents. Households were sampled without replacement on a daily basis such that no household was observed more than once per day.

The example dataset focuses on the activities of males because there is a pronounced sexual division of labor in this community, and women rarely engage in several of the activities in our ethogram, such as agriculture, collecting firewood, and hunting. (There is no *a priori* reason why females would need to be excluded, however, as the individuals’ sex could be included as a fixed effect predictor and the number of response categories could also be expanded to include female-specific behaviors, such as childcare.) For this analysis of male activities, Table 1 describes the behaviors that characterize men’s work in this setting. The reference category in this analysis consists of non-work activities, including sleeping, idleness, socializing, and recreation. We choose this as the reference category partly because individual-level correlations between this and other categories are substantively less interesting than correlations between the remaining work-related activities.

**Table 1:** Description of activities that comprise the response categories

Response	Description
(1) Agriculture	Activities including clearing fields, planting, weeding, and harvesting crops
(2) Domestic chores	Cooking, laundering clothes, cleaning the residence, bringing water, etc.
(3) Staying at finca	Extended time at makeshift upstream residences, involving overnights
(4) Firewood	Either collecting firewood from forest or chopping firewood in community
(5) Fishing	Excursions specifically devoted to fishing
(6) Gold panning	Either preparing sites or actively panning for gold in streams around community
(7) Hunting	Excursions devoted specifically to hunting activities, not opportunistic hunting
(8) Livestock	Either direct care of domestic animals or preparation of pastures and shelters
(9) Manufacture	Constructions of items such as dugout canoes, residences, or homemade tools
(10) Miscellaneous work	Involves community labor, errands, providing routine assistance to others
(11) School	Attending school as a student
(12) Steady work	Regular employment as a schoolteacher, contract worker, or project assistant
(13) Wage labor	Working for pay locally, including clearance of fields and construction tasks
(14) Reference	Non-work reference level for idleness, sleeping, leisure, church, socializing, etc.

Table 2 presents descriptive statistics on the fixed effect covariates. Our initial focus is on age, following predictions that individuals behave in ways that capitalize on their strength and skills at different ages. For example, adolescent males are expected to focus on activities that require relatively little strength and skill, such as fishing and livestock care. As they mature, adult men are predicted to focus on high-strength, high-skill activities, such as hunting and clearing fields. Older men whose strength has declined are expected to transition into activities such as manufacturing tools and routine agricultural activities. We fit first- and second-order polynomials of age to allow the propensity for certain kinds of work to increase and decline across the lifespan (and vice versa).

**Table 2:** Predictor variable names, descriptions, and summary statistics

Variable	Description	Mean	Std dev.
Age	Age in years of observed individuals	31.13	15.63
Wealth	Log-transformed value of household possessions (measured in Nicaraguan currency)	8.60	0.93
House size	Number of residents in the household of the observed individual at the time of the observation	8.16	3.04
Sunday	Binary variable to denote observations that occurred on Sunday	.12	
Saturday	Binary variable to denote observations that occurred on Saturday	.15	
Time of day	Proportional variable that denotes that percentage of a 24-h day that had elapsed at the time of observation	0.49	0.15
Monthly rainfall	Average monthly rainfall (mm) for the month in which the observation occurred	222.10	112.19

Other covariates include standard demographic variables, such as household wealth and household size. The remaining variables reflect the temporal patterning of work that characterizes this setting. For instance, members of this community work less on Sunday because they observe the Sabbath, and Saturday is regarded as an ideal day for hunting and collecting firewood. Although non-human animals do not necessarily follow such calendars, we note that behavioral ecologists may wish to make analogous categorical distinctions, such as distinctions between ruminants’ rutting periods and other times (e.g., Miquelle 1990). Regarding circadian variation, behavioral ecologists regularly control for “time of day” in their statistical models (e.g., Hill et al. 2003), which we include as a proportional variable with first- and second-order polynomials given our expectation that certain work activities are particularly common at midday. Finally, there is often seasonal variation in behavior (e.g., Wittemyer et al. 2007), and several subsistence activities in Nicaragua depend partly on rainfall and river levels, such as agriculture and fishing. As a control variable, we use measurements of average monthly rainfall at a nearby weather station (Koster et al. 2016). All continuous variables are *z*-score standardized, both to facilitate estimation using *RStan* and to facilitate interpretation and the generation of predictions from the posterior samples.

## Estimation

Multilevel, multinomial logistic regression models are not routinely implemented in several statistical packages that are commonly used by behavioral ecologists, such as *lme4* (Bates et al. 2015). Furthermore, for high-dimensional multilevel models, Markov chain Monte Carlo (MCMC) estimation is generally superior to maximum likelihood methods (see Bolker et al. 2013). With the advent of packages that facilitate MCMC estimation, behavioral ecologists are better able to specify models that meet the challenging nature of their data structures.

For this analysis, we demonstrate the use of *RStan*, which uses Hamiltonian Monte Carlo (HMC) methods that depart from the Gibbs samplers and Metropolis-Hastings algorithms that were implemented in earlier packages, such as BUGS and MLwiN (Lunn et al. 2000; Browne and Rasbash 2009). Hamiltonian Monte Carlo estimation has clear advantages for complex models, and we refer readers to McElreath (2015) for a helpful overview of the method, including advice on convergence diagnostics and interpretation. Despite the advantages, however, HMC methods alone are not a panacea for all challenges of estimation. Care must still be taken to choose a parameterization of the model that mixes well. In this case, we rely on a non-centered parameterization of the varying effects, using a Cholesky factorization of the variance-covariance matrices (McElreath 2015:405). To further promote good mixing of the HMC chains, we supply weakly informative priors for the fixed effect parameters and variance-covariance matrices. These priors prevent the sampler from considering highly implausible values, and the priors are weak because they otherwise assume that zeroes represent the highest probabilities for parameters (including correlations). When the posterior distributions of parameters are centered around non-zero values, it is because the empirical data provides contravening evidence to the weak prior. When the data are not informative about the parameters, by contrast, the model will default to the weak prior.

We present four models, which vary in their random effects structure and the inclusion of fixed effect covariates. That is, the simpler models include only random effects for the observed individuals while expanded models add random effects for household and month. Then for each of these random effect structures, we present models with and without the fixed effects, which receive an *F* suffix to help users navigate between the models in the paper and the supplemental script.

Supplemental Folder 1 includes the data and annotated script that we use for specifying models and processing the posterior samples. To complement *RStan*, the *rethinking* package includes convenience functions for preparing data, summarizing the posterior, and plotting model predictions. For all model fitting, we specify three chains of 2000 iterations, half of which are devoted to the warm-up. Model diagnostics indicate adequate mixing of the chains.

For each model, we calculate the Widely Applicable Information Criterion (WAIC), which has fewer restrictive assumptions than the Deviance Information Criterion (DIC), a commonly used analogue (McElreath 2015). As with other information criteria, lower values indicate preferred models that successfully balance predictive accuracy against model complexity and overfitting. Although this paper does not emphasize a model comparison approach, in turn fitting many candidate models to determine which set of parameters best balances the bias-variance trade-off (Symonds and Moussalli 2011), such approaches are nonetheless possible with multinomial logistic regression models. In this paper, the WAIC is included partly to familiarize behavioral ecologists with this metric.

## Results: WAIC

Of the four models, the most complex model (*mfit_ihmF*) receives the strongest support from the WAIC comparison (Table 3; see also Supplemental Fig. 1). This model includes the full set of fixed effects and random effects for individuals, households, and the months in which observations occurred. In addition to having the lowest WAIC, this model also receives full support from the comparison of WAIC weights, which are analogous to AIC weights (Johnson and Omland 2004). The WAIC weight of a model is interpretable as the probability that the model will make the best predictions on new data relative to the other models under consideration.

**Table 3:** Model comparison using WAIC

Model	WAIC (SE)	Effective parameters	ΔWAIC (SE)	Weight
*mfit_ihmF*	8447.0 (122.72)	362.4		1
*mfit_iF*	8721.6 (123.68)	284.3	274.6 (31.83)	0
*mfit_ihm*	9267.0 (113.26)	324.5	820.0 (52.34)	0
*mfit_i*	9574.4 (112.97)	231.5	1127.4 (60.67)	0

Lower values indicate preferable models. The weight of a model is its Akaike weight, interpretable as the probability that a candidate model will make superior predictions on new data

## Results: interpreting the variance/covariance of the “intercept-only” model (mfit_i)

A common approach to multilevel modeling analyses is to begin with a model that includes the random effects but no fixed effects other than the covariates. These models provide insight into the hierarchical data structure and the correlations among random effects. The first model therefore includes only the intercepts and the random effects (i.e., varying intercepts) for the observed individuals. The coefficients for the intercepts are presented in Supplemental Table 1, de-emphasized here because their predicted probabilities correspond almost identically to the corresponding percentages from the empirical data.

For each categorical response, the variance of the random effects is reported in the first column of Table 4, which includes the variance estimates from all fitted models. The extent of the individual-level variance is heterogeneous across the responses. Behaviors that exhibit relatively low variance, such as *agriculture* and *firewood*, are largely compulsory for all individuals in this setting. By contrast, high-variance activities such as *gold panning* and *steady work* represent the economic specializations of a subset of men. Another high-variance activity, *school*, is limited to adolescents.

**Table 4:** Variance estimates of the random effects in the four models presented in this paper. The reported quantities are the standard deviations of the random effects while the values in parentheses are the standard deviations of these quantities in the posterior samples

	Individual				House		Month	
	*mfit_i*	*mfit_iF*	*mfit_ihm*	*mfit_ihmF*	*mfit_ihm*	*mfit_ihmF*	*mfit_ihm*	*mfit_ihmF*
1. Agriculture	.67 (.10)	.50 (.11)	.62 (.11)	.41 (.14)	.24 (.15)	.30 (.15)	.52 (.16)	.46 (.15)
2. Domestic	1.08 (.28)	.87 (.33)	.80 (.33)	.62 (.36)	.73 (.41)	.62 (.38)	.28 (.22)	.23 (.19)
3. Finca	1.80 (.31)	1.89 (.33)	1.47 (.37)	1.58 (.44)	1.09 (.53)	1.30 (.69)	1.00 (.30)	1.11 (.32)
4. Firewood	.29 (.19)	.23 (.17)	.25 (.18)	.22 (.17)	.31 (.20)	.28 (.20)	.79 (.25)	.74 (.25)
5. Fishing	.90 (.28)	.89 (.30)	.38 (.27)	.34 (.27)	.98 (.36)	1.00 (.37)	.71 (.37)	.71 (.35)
6. Gold	2.23 (.38)	2.28 (.43)	1.60 (.39)	1.39 (.44)	1.64 (.62)	1.76 (.57)	.56 (.19)	.32 (.20)
7. Hunting	1.31 (.27)	1.29 (.30)	.92 (.31)	.80 (.43)	.96 (.47)	1.00 (.53)	.23 (.18)	.35 (.26)
8. Livestock	.70 (.36)	.74 (.40)	.41 (.32)	.46 (.35)	.72 (.37)	.83 (.41)	1.28 (.73)	1.14 (.67)
9. Manufacture	1.02 (.19)	.81 (.19)	.87 (.22)	.37 (.26)	.47 (.28)	.74 (.27)	.27 (.18)	.24 (.17)
10. Other work	.82 (.20)	.57 (.24)	.73 (.21)	.42 (.25)	.32 (.22)	.38 (.25)	1.59 (.57)	1.38 (.46)
11. School	1.64 (.35)	.81 (.38)	1.68 (.41)	.43 (.33)	.60 (.50)	.59 (.40)	2.36 (.99)	1.78 (.77)
12. Steady work	2.91 (.63)	2.85 (.56)	2.84 (.60)	2.80 (.57)	.84 (.81)	.71 (.62)	.32 (.20)	.30 (.19)
13. Wage	1.10 (.21)	.53 (0.24)	.83 (.26)	.31 (.22)	.73 (.32)	.67 (.24)	1.06 (.34)	1.01 (.31)

The lower half of the matrix in Table 5 presents the correlations of the individual-level random effects across the 13 behavioral responses (other than the reference category). Most correlations are modest and lacking strong statistical support. However, some moderate correlations are evident. Males with a high relative risk of *agriculture* also have relatively high random intercepts for *manufacture* (*ρ*_1 ,9_ = 0.39) and *other work* (*ρ*_1 ,10_ = 0.37). These correlations plausibly relate to age given the aforementioned prediction that older men dedicate themselves to high-skill, low-strength activities. Similarly, the negative correlation between *school* and *wage labor* (*ρ*_11 ,13_ =  − 0.48) relates to the unavailability of adolescent males for wage labor opportunities on weekdays when they are attending school.

**Table 5:** Correlations of individual-level random effects across responses

	(1)	(2)	(3)	(4)	(5)	(6)	(7)	(8)	(9)	(10)	(11)	(12)	(13)
1. Agriculture		.00 (.21)	.**41 (.15)**	.05 (.24)	.07 (.21)	.01 (.17)	.04 (.20)	− .01 (.23)	.30 (.18)	.14 (.23)	.13 (.22)	− .24 (.18)	.04 (.22)
2. Domestic	.02 (.18)		.07 (.19)	− .02 (.25)	.15 (.22)	− .02 (.21)	.13 (.21)	.14 (.23)	− .15 (.22)	.06 (.23)	.06 (.24)	− .02 (.22)	.03 (.23)
3. Finca	.24 (.15)	.14 (.17)		.00 (.24)	.05 (.19)	.18 (.15)	**.40 (.17)**	.06 (.21)	− .10 (.18)	.20 (.21)	.13 (.22)	− .23 (.16)	− .02 (.20)
4. Firewood	.12 (.23)	.03 (.23)	− .01 (.23)		.07 (.24)	.06 (.24)	.00 (.24)	.01 (.25)	.09 (.25)	− .01 (.24)	.04 (.25)	− .03 (.24)	− .04 (.25)
5. Fishing	− .10 (.19)	.10 (.21)	.01 (.18)	.06 (.24)		.23 (.20)	.21 (.21)	.07 (.23)	.10 (.21)	.07 (.23)	− .03 (.22)	− .07 (.21)	.18 (.22)
6. Gold	.03 (.16)	− .11 (.19)	.19 (.14)	− .03 (.24)	.21 (.19)		**.35 (.16)**	.15 (.20)	− .04 (.18)	.10 (.20)	− .15 (.23)	**− .36 (.17)**	.13 (.20)
7. Hunting	− .02 (.17)	.14 (.19)	**.44 (.15)**	− .11 (.23)	.07 (.20)	**.39 (.15)**		.14 (.22)	.02 (.20)	.24 (.21)	− .05 (.24)	− .13 (.19)	.13 (.21)
8. Livestock	.03 (.22)	.17 (.22)	.09 (.21)	.03 (.24)	.02 (.22)	.16 (.21)	.17 (.21)		− .09 (.23)	.18 (.24)	.07 (.24)	− .01 (.22)	− .06 (.23)
9. Manufacture	**.39 (.15)**	− .13 (.19)	− .12 (.16)	.03 (.23)	− .06 (.20)	.09 (.16)	.16 (.17)	.00 (.22)		.00 (.22)	− .02 (.22)	.03 (.19)	.02 (.21)
10. Other work	**.37 (.17)**	.09 (.21)	.16 (.17)	− .03 (.24)	− .09 (.22)	.15 (.18)	.31 (.18)	.23 (.23)	.30 (.18)		.12 (.24)	− .14 (.22)	− .03 (.24)
11. School	− .21 (.17)	.21 (.18)	.21 (.17)	.08 (.23)	.14 (.19)	− .24 (.18)	− .07 (.19)	.04 (.22)	− .33 (.18)	− .18 (.20)		− .09 (.23)	− .21 (.24)
12. Steady	− .19 (.16)	.02 (.21)	− .14 (.15)	− .08 (.23)	− .12 (.21)	− .19 (.17)	.09 (.18)	.05 (.22)	.17 (.17)	.04 (.20)	− .18 (.19)		− .01 (.21)
13. Wage	.27 (.15)	− .01 (.19)	− .06 (.15)	− .11 (.23)	.08 (.20)	**.31 (.15)**	.22 (.17)	− .05 (.21)	.28 (.16)	.23 (.18)	**− .48 (.16)**	.14 (.17)	

The reported means are from the posterior samples (standard deviation in parentheses). Parameters in bold represent estimates whose 96% credible intervals do not include zero. The bottom half of the matrix depicts correlations from the intercept-only model (*mfit_i*). The top half of the matrix details correlations from the model with fixed effects, but not additional random effects (*mfit_iF*)

## Results: interpreting the variance/covariance of the fixed effects model (mfit_iF)

In addition to the random effects for individuals, our second model includes the predictor variables, but before addressing their interpretation, we revisit the variances and correlations across responses. As a cautionary note, Snijders and Bosker (2012, 307–309) emphasize that the inclusion of fixed effects potentially raises the higher-level variance in multilevel models because whereas the lowest-level variance is fixed, the scale of the higher-level variance is arbitrary. Unlike linear mixed models of normally distributed response variables, in which comparisons of variance across models can facilitate “percentage of variance explained” calculations (similar to conventional *R*-squared measures), changes in generalized linear models merit caution because the inclusion of fixed effects may have unanticipated effects on the variance. Thus, although the variance estimates increase for several of the responses (e.g., *finca*), such changes cannot be conclusively regarded as a by-product of a correlation between predictor variables and random effects (see Gelman and Hill 2007, 480–481). Despite those caveats, it is evident that the fixed effects account for substantial individual-level variance in several behavioral categories, such as *school* and *wage labor* (Table 4).

The standardized correlations across the behavioral categories are not equally sensitive to the arbitrary scaling of the variance. A comparison of the correlations from the model with fixed effects (*mfit_iF*) to the earlier “intercept-only” model shows that several correlations exhibit moderate effects (see the top half of Table 5). For example, there are positive correlations between *hunting* and *finca* and *gold panning*, respectively, which remain robust in both models. As a possible explanation, these correlations suggest a peripatetic lifestyle in which some men frequently sojourn through the forest, staying at their makeshift homes and using their hunting expeditions as an opportunity to evaluate the streams they encounter as possible sources of gold.

Not all correlations remain robust across models. For example, the aforementioned correlation between *agriculture* and *manufacture* is now weaker (*ρ*_1 ,9_ = 0.30). Such changes relate to the impact of fixed effects on the estimated random effects. A misconception is that random effects are static across models and that, for instance, an individual with a high random intercept in the initial model will have a similarly high random intercept in all subsequent models. This misconception may stem from the nomenclature and the emphasis on “intercepts.” Instead, random effects are estimated in relation to the effects of all covariates (including the intercept). An individual may exhibit a high propensity for a behavior relative to the fixed part prediction, yielding a positive random effect, but with the addition of further covariates, the positive random effect may change its magnitude and sign. Thus, the correlations of the random effects must be considered in relation to the predictor variables in the model.

For similar reasons, correlations that were weak in the “intercept-only” model may now exhibit stronger effects. For example, the negative correlation between *gold panning* and *steady work* increased from −0.19 to −0.36, evidently because of the relationship between these behaviors and the individuals’ age and household wealth (note that adolescent males do comparatively little of either behavior). Controlling for these variables, males who conduct more steady work are less frequently observed to be gold panning.

## Results: interpreting the coefficients and predicted probabilities of the fixed effects model (mfit_iF)

Table 6 presents coefficients of the fixed effects, which are interpreted as the effect of a one-unit increase in the predictor on the log-odds of exhibiting behavior *k* instead of the reference category, conditional on the other parameters. The focus on this contrast is important to reiterate because the coefficients are not straightforward indicators of the effect of a predictor on the probability of doing behavior *k*. As noted by Retherford and Choe (1993, 153), there are scenarios in which covariates predict higher probabilities of a behavior despite a coefficient that is zero or even the opposite sign.

**Table 6:** Posterior means (standard deviations in parentheses) of fixed effects in models *mfit_iF* and *mfit_ihmF*, respectively

	Agriculture	Domestic	Finca	Firewood	Fishing	Gold	Hunting	Livestock	Manufacture	Other work	School	Steady work	Wage labor
*mfit_iF*													
Intercept	.21 (.16)	**− 3.30 (.38)**	**− 1.36 (.41)**	**− 2.28 (.26)**	**− 2.91 (.39)**	− .95 (.50)	**− 1.96 (.39)**	**− 3.34 (.41)**	**− 1.07 (.26)**	**− 2.16 (.28)**	**− 3.73 (.50)**	**− 2.29 (.62)**	**− .71 (.22)**
Age	**.70 (.12)**	− .05 (.25)	.11 (.35)	.07 (.16)	− .26 (.27)	.77 (.44)	.50 (.31)	.31 (.28)	**1.05 (.21)**	**1.03 (.23)**	**− 1.83 (.44)**	.75 (.59)	**1.00 (.19)**
Age^2^	− .12 (.10)	− .05 (.25)	− .29 (.32)	.22 (.13)	− .03 (.26)	**− 1.31 (.43)**	**− 1.00 (.31)**	− .18 (.25)	**− .59 (.18)**	**− .44 (.18)**	.26 (.47)	**− 2.03 (.64)**	**− .77 (.17)**
Wealth	− .14 (.10)	.34 (.22)	.35 (.30)	− .04 (.14)	− .23 (.24)	− .10 (.36)	.55 (.28)	.35 (.25)	− .02 (.17)	.10 (.17)	.15 (.25)	.32 (.46)	**− .36 (.14)**
House size	**− .19 (.10)**	− .43 (.21)	**− .36 (.15)**	− .15 (.14)	− .06 (.22)	.03 (.20)	− .37 (.23)	.14 (.24)	− .04 (.15)	− .16 (.17)	− .30 (.20)	.29 (.30)	− .15 (.14)
Sunday	**− 3.45 (.42)**	− .20 (.39)	**− 1.44 (.27)**	**− 2.33 (.59)**	**− 1.73 (.55)**	**− 2.94 (.48)**	**− 1.50 (.59)**	− 1.06 (.55)	**− 2.25 (.42)**	− .08 (.31)	**− 2.23 (.59)**	**− 2.37 (.44)**	**− 2.93 (.53)**
Saturday	.31 (.16)	.37 (.38)	.17 (.20)	**.73 (.27)**	.42 (.35)	− .39 (.27)	**2.23 (.28)**	.15 (.49)	− .17 (.28)	− .27 (.40)	**− 1.83 (.66)**	− .42 (.31)	**− 1.13 (.37)**
Time	.07 (.07)	.05 (.14)	− .09 (.08)	− .03 (.13)	− .17 (.15)	− .04 (.12)	− .36 (.20)	− .27 (.19)	− .17 (.10)	**.49 (.15)**	.26 (.19)	− .01 (.12)	**.25 (.12)**
Time^2^	**− 1.14 (.09)**	− .29 (.17)	**− .50 (.09)**	**− .71 (.15)**	**− .54 (.17)**	**− 1.31 (.13)**	**− 1.49 (.23)**	− .34 (.21)	**− .66 (.12)**	**− .71 (.16)**	**− 1.06 (.21)**	**− .91 (.13)**	**− .84 (.13)**
Rainfall	**− .24 (.07)**	.23 (.15)	− .12 (.08)	.00 (.12)	− .13 (.16)	.**35 (.10)**	.04 (.15)	.19 (.19)	.15 (.10)	.**39 (.13)**	.20 (.15)	− .02 (.11)	**− .24 (.10)**
*mfit_ihmF*													
Intercept	.26 (.24)	**− 3.37 (.40)**	**− 1.48 (.57)**	− **2.23 (.37)**	**− 2.92 (.49)**	− .94 (.53)	**− 2.13 (.43)**	**− 3.18 (.62)**	**− 1.07 (.29)**	**− 2.05 (.51)**	**− 3.25 (.76)**	**− 2.20 (.65)**	**− .92 (.40)**
Age	**.70 (.12)**	− .13 (.25)	.41 (.39)	.04 (.18)	− .32 (.25)	.67 (.41)	.56 (.30)	.25 (.28)	**.97 (.19)**	**1.02 (.22)**	**− 1.97 (.45)**	.77 (.58)	.**97 (.20)**
Age^2^	− .12 (.11)	.10 (.25)	− .46 (.31)	.22 (.14)	.11 (.24)	**− 1.24 (.38)**	**− .87 (.29)**	− .15 (.25)	**− .63 (.17)**	**− .42 (.18)**	.38 (.46)	**− 1.98 (.64)**	**− .68 (.17)**
Wealth	− .14 (.11)	.35 (.24)	.26 (.37)	− .04 (.16)	− .27 (.30)	− .26 (.43)	.48 (.32)	.41 (.30)	− .02 (.18)	.09 (.19)	.20 (.26)	.38 (.46)	**− .45 (.18)**
House size	− .19 (.10)	− .36 (.23)	**− .61 (.18)**	− .11 (.15)	.04 (.26)	.15 (.23)	− .43 (.27)	.29 (.28)	− .09 (.17)	− .11 (.18)	− .29 (.22)	.33 (.32)	− .06 (.18)
Sunday	**− 3.45 (.42)**	− .20 (.37)	− **1.53 (.26)**	**− 2.21 (.59)**	**− 1.71 (.57)**	− **2.94 (.49)**	**− 1.52 (.58)**	**− 1.05 (.55)**	**− 2.25 (.45)**	.09 (.32)	**− 2.13 (.62)**	**− 2.35 (.44)**	**− 2.85 (.54)**
Saturday	.**33 (.16)**	.37 (.38)	.14 (.21)	**.75 (.28)**	.43 (.34)	− .43 (.28)	**2.23 (.29)**	.16 (.47)	− .16 (.28)	− .28 (.42)	**− 1.86 (.63)**	− .42 (.32)	**− 1.18 (.38)**
Time	.08 (.07)	.05 (.14)	− .09 (.08)	− .02 (.14)	− .19 (.17)	− .04 (.11)	− .34 (.20)	− .25 (.20)	− .17 (.10)	**.48 (.16)**	.23 (.19)	− .02 (.12)	**.28 (.12)**
Time^2^	**− 1.17 (.09)**	− .30 (.16)	**− .50 (.09)**	**− .71 (.15)**	**− .54 (.18)**	− **1.30 (.14)**	**− 1.49 (.23)**	− .35 (.21)	**− .66 (.12)**	**− .74 (.17)**	**− 1.07 (.21)**	**− .93 (.14)**	**− .84 (.13)**
Rainfall	− .28 (.15)	.24 (.18)	− .11 (.33)	− .05 (.25)	− .13 (.27)	**.33 (.14)**	.04 (.20)	.25 (.41)	.15 (.13)	.37 (.41)	.25 (.50)	− .02 (.15)	− .23 (.31)

Parameters in bold represent estimates whose 96% credible intervals do not include zero. Note that all continuous predictors have been *z*-score standardized relative to their values in Table 1

Such insights underscore the importance of computing the predicted probabilities. In the script that accompanies this paper, we include a function that assists with these calculations. Directly analogous to the *link* function in the *rethinking* package (McElreath 2015), this function is specific to multinomial logistic models. Dubbed *link.mn* in the accompanying script, this function allows users to supply customized values for the covariates, which are then multiplied by the corresponding coefficients for each sample in the posterior. Complementary functions then summarize the means and prediction intervals of the values generated by the *link.mn*, which relies on the softmax function to normalize the predicted *K* probabilities to sum to 1. The function provides the option to incorporate the random effects or to calculate probabilities only from the fixed effects, which is the method used in this example.

**Fig. 1:**
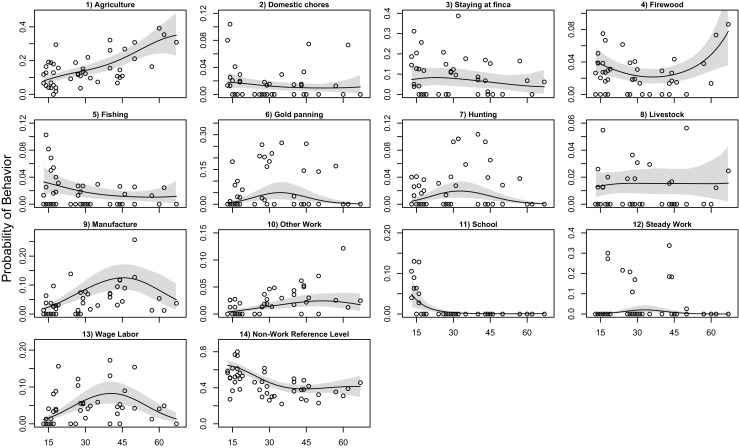
Model predictions of response behaviors as a function of age. Predictions assume a time of 8:00 a.m. on a weekday. All other covariates are held at the sample mean. The shaded regions are the 89% percentile intervals, as calculated from the posterior samples of model *mfit_iF*

Figure 1 depicts the predicted probabilities for the 14 behavioral categories across the range of observed ages in the empirical data. For these predictions, only age varies while all other coefficients are held at a constant value. Noteworthy results are that time allocation to *agriculture* increases across the lifespan whereas other behaviors are more common in middle age, such as *gold panning*, *hunting*, and *wage labor*. These results are largely consistent with the aforementioned hypothesis from life history theory, but contrary to predictions, *manufacture* likewise exhibits a peak in middle age.

**Fig. 2:**
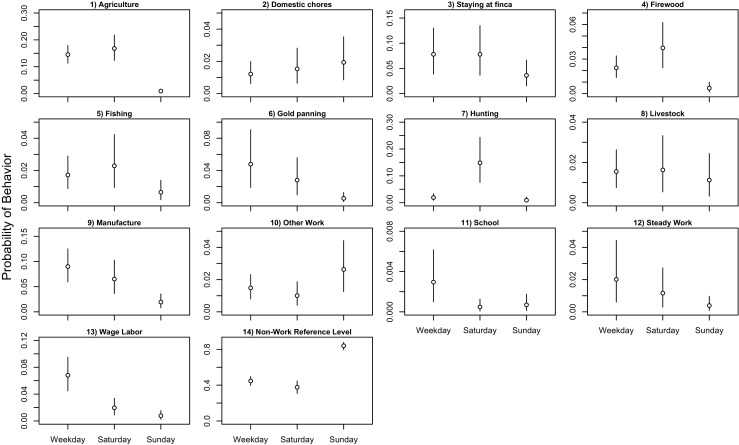
Model predictions of response behaviors as a function of day. Predictions assume a time of 8:00 a.m. while all other covariates are held constant at the sample mean. The confidence intervals are the 89% percentile intervals, as calculated from the posterior samples of model *mfit_iF*. Note the similarity of ratios between *other work* (*k* = 10) and the reference level (*k* = 14), which was addressed as an example in the text of the manuscript

Predicted probabilities can also be generated for categorical predictors. Figure 2 shows the predicted probabilities for weekdays, Saturday, and Sunday. On Saturdays, hunting behavior increases dramatically, suggesting that hunting trips are motivated in part by cultural norms rather than dynamic responses to short-term nutritional needs or favorable conditions for foraging (Stephens and Krebs 1986; Sosis 2002). On Sundays, the results indicate that several work activities are less common, particularly laborious activities that require sojourns away from the residential site (e.g., *agriculture*, *firewood*, *gold panning*, *hunting*).

On the other hand, *other work* is evidently more common on Sundays than other days. This example is instructive because of the evidence that the coefficient itself is near zero (*β* = − 0.08), paralleling the earlier note about possible misinterpretations of parameters in multinomial models. That is, *other work* increases in frequency on Sundays, but so does the reference category, and so the ratio of *other work* to the non-work reference category remains largely constant across weekdays, Saturdays, and Sundays. Hence the coefficients are effectively indistinguishable from zero even though there are evident differences in the probability of this behavior on different days.

Researchers are often interested in testing for differences among multiple categorical predictor variables (e.g., the post hoc tests in conventional ANOVA models). Continuing this example, there might be substantive interest in testing for differences between weekdays, Saturdays, and Sundays on the probability of *other work*. The prediction intervals depicted in Fig. 2 are inadequate for this purpose because they incorporate uncertainty from all of the parameters in the model, not just the contrasts of interest (i.e., the overlapping prediction intervals are not an indication that there are no distinguishable differences between *other work* on different days).

To test for differences among categorical predictors, one recommended strategy is to calculate the differences between each contrast for each sample in the posterior, then use the distribution of those differences for inference. Supplemental Fig. 2 shows these differences, which reveal a consistent, albeit modest increase in the probability of *other work* on Sunday. The predictions offer less confidence that there is a difference in the probability of *other work* on weekdays and Saturdays.

As a final set of predictions from this model, Supplemental Fig. 3 shows that the frequency of several behaviors is dependent on the time of day. In general, work activities increase until midday, then decline as dusk approaches.

## Results: interpreting the models with additional random effects (mfit_ihm and mfit_ihmF)

The impetus for including random effects for households and months partly relates to the significant effects exhibited by fixed effects that are defined at these levels of the data structure. For instance, in the preceding model (*mfit_iF*), the relative risk of *wage labor* declines with increased *wealth*, and the relative risk of *agriculture* declines with *rainfall*. That model, however, does not account for the clustering of the data by household and month. A distinct advantage of multilevel modeling is that higher-level predictors can be included in models while the corresponding random effects adjust for the clustering (Goldstein 2011).

To generate predictions across the range of the fixed effects, we modify the *link.mn* function to account for the additional random effects structure. Based on the model that includes the additional random effects and the full set of fixed effects (*mfit_ihmF*), simulated predictions for *wealth* show that individuals from wealthier households tend to conduct less *wage labor*, but wealth is not a strong predictor of other behaviors (Supplemental Fig. 4). Similarly, few behaviors seem contingent on *house size* (Supplemental Fig. 5). Finally, there is a tendency for *agriculture* to decline with *monthly rainfall* while *gold panning* increases, but the effects are relatively modest (Supplemental Fig. 6). In general, the predictions of the fixed effects change little when incorporating the additional random effects.

The extended models continue to include the variance/covariance matrix for the individual-level random effects, but with the inclusion of the household-level random effects, the interpretation has changed. In this parameterization of the model, the individual-level random effects are interpretable as the deviation from the household-level average (i.e., the household’s random effect). The variance estimates now reflect the within-house variation among individuals, not the variation across individuals in the population. The correlations across behavioral responses therefore provide less insight into individual-level trade-offs, though they remain largely consistent with earlier inferences (see Supplemental Tables 2–4 for all correlation matrices pertaining to models *mfit_ihm* and *mfit_ihmF*).

With a data structure that includes only 45 males distributed among 25 households, the household-level random effects are estimated imprecisely. There is little inferential insight to be gained from considering the variance estimates or their correlations. Regarding the random effects for month, some behaviors exhibit substantial variance across months, and this variance is only moderately explained by the fixed effects (see again Table 4). Predictor variables such as *rainfall* explain relatively little behavioral variation, for instance. Other variables could potentially be included in the models to account for this temporal heterogeneity, such as a binary variable to denote extended school vacations, but we do not pursue those extensions here.

## Discussion

This paper describes a multilevel multinomial behavior model that employs principles of generalized linear mixed models for the analysis of observational data. A review of the behavioral ecological literature suggests that this modeling strategy has been used rarely, appearing intermittently only recently. However, because multinomial models are well suited to the structure of observational data collected via scan sampling methods, these models merit strong consideration as the default choice for future studies. Instead of relying on aggregations across behavioral categories or within observed individuals, we use unaggregated data to model the probability of observing behavior *k* by individual *i* at time *t*. These models therefore permit the inclusion of time-varying covariates (e.g., seasonal heterogeneity) while relying on random effects to address the pseudoreplication and the imbalanced sampling of individuals that typify field research. Beyond treating this individual-level variance as a nuisance to be remedied, the models presented here show how correlated random effects across the response categories can elucidate behavioral dimensions and trade-offs that interest behavioral ecologists. Because of the potentially valuable inferences afforded by these correlations, explaining the mechanics and interpretation of the correlated random effects has been the primary emphasis of this paper.

The multinomial format does not fully relieve researchers of important decisions about ethograms and the coding of behavioral categories. In principle, there is not a maximum number of response categories that can be accommodated in multinomial models. In practice, however, when there are few observations of a particular behavior, then the posterior distribution will merely reflect the model’s prior for those rare behaviors, suggesting possible benefits for combining behaviors from the original coding scheme. For the dataset used in this paper, for instance, the original ethogram distinguished between different components of livestock care, which were subsequently combined because of the rarity of these behaviors. There are few clear solutions to automate this process, and we anticipate that similar decisions about the definition of categories will largely depend on the researchers’ familiarity with the behaviors and the population being studied.

In addition to the basic multinomial modeling approach presented here, possible extensions include options that characterize generalized linear mixed models more generally. Whereas the models in this paper focused only on varying intercepts (individuals, households, and months), it would also be possible to estimate varying slopes for covariates in the models (Leckie and Goldstein 2015). For example, if a longitudinal dataset were to include observations of individuals at different ages, then the effect of age could be allowed to vary across individuals. The expansion of the variance-covariance matrix to accommodate these varying slopes would result in additional correlations that could reveal the extent to which time allocation at older ages is contingent on behavioral strategies earlier in life.

A final methodological consideration pertains to temporal autocorrelation. In many empirical settings, observations conducted at temporally proximate intervals are likely to document similar patterns of behavior. For the example dataset, we accounted for temporal variation in different ways. For behavioral variation related to time of day, we used first- and second-order polynomial effects, which account for the gradual increase and subsequent decline of work activities throughout the day (see also Wright et al. 2014). For variation related to the month in which activities were observed, we used conventional random effects for month, paralleling similar longitudinal studies that use random effects for calendar date, month, or year (Griesser and Nystrand 2009; McElreath and Koster 2014; Requena and Machado 2015; Kerhoas et al. 2016). This latter approach has limitations, namely, that it implies an exchangeable correlation structure in which observations within a cluster are equally unrelated to all other clusters. In many cases, though, researchers may anticipate that behaviors exhibited at proximate times are more similar than behaviors across temporally disparate intervals. For example, behaviors in April and May are potentially more similar than behaviors occurring 5 or 6 months apart.

Similar concerns have recently motivated statistical approaches that address temporal autocorrelation (Fürtbauer et al. 2011; Nakayama et al. 2016). In addition to these alternatives, multinomial models potentially benefit from the development of Gaussian process regression (Rasmussen and Williams 2006), as summarized by McElreath (2015) and facilitated by the implementation of the Gaussian process in the *RStan* package. Instead of discrete boundaries between categories, such as discrete months or households, Gaussian process models rely on a matrix of distances between pairs of observations (e.g., the amount of time between the respective observations). Thus far, extensions of the approach to multinomial logistic models have largely been limited to the machine learning literature (e.g., Chai 2012). As behavioral ecologists contend with temporal autocorrelation both across and within individuals, however, we anticipate promising alternatives that incorporate principles and methods of Gaussian process regression. The caveat is that statistical models by ecologists can be unnecessarily complex (Murtaugh 2007; Cressie et al. 2009), and the sample sizes that typify observational studies may not accommodate the added complexity.

In terms of substantive contributions, the results of this paper provide intermediate support for the prevailing hypothesis that heterogeneous combinations of strength and skill across the lifespan predict variation in behavioral strategies (Gurven and Kaplan 2006). The behavior that most closely adheres to the prediction is *agriculture*, which becomes more frequent later in life, arguably because it requires advanced botanical knowledge but not strenuous activity. Other behavioral categories roughly conform to predictions. Some activities, such as *fishing* and *domestic chores*, are modestly more frequent among adolescents while strenuous, high-skill activities such as hunting and gold panning are more common among middle-aged men. Overall, however, age explains only a limited amount of the variation in the behavioral outcomes, and many behaviors exhibit considerable individual-level heterogeneity. Given the intellectual and capital investments required of some subsistence activities, this variation could potentially be explained by long-term returns to specialization (Schniter et al. 2015). More generally, at a time when individual-level behavioral variation and personality are attracting attention from behavioral ecologists (Bell et al. 2009; Beleyur et al. 2015), the present statistical approaches align with efforts to use multilevel models to quantify the repeatability of behavior (Nakagawa and Schiezeth 2010; Dingemanse and Dochtermann 2013).

## Conclusion

This paper emphasizes a modeling approach that leverages correlated random effects to gain insight into the surprisingly elusive question of how time spent in one activity precludes time allocation to other activities. Behavioral ecologists have theorized at length about these trade-offs, but the analysis of observational data has been limited by prevailing methods that require aggregations of the original data. The development of statistical tools can stimulate new theorizing (Gigerenzer 1991), and much like the proliferation of multiple regression software formerly led to broader multicausal hypothesizing in the behavioral sciences, the availability of multinomial models for observational data potentially revitalizes theorizing about trade-offs and predictors of behavior. The accessibility of statistical software for estimating models is essential, and this paper benefits from the development of the *RStan* package and its Hamiltonian Monte Carlo algorithm. As a complement to the coding script that accompanies this paper, there is potential for the preparation of additional convenience functions to facilitate analyses that rely on the basic modeling framework that we have espoused for behavioral data.

## Electronic supplementary material

ESM 1 (PDF 1304 kb)

ESM 2 (DOCX 65 kb)

ESM 3 (ZIP 43.5 kb)
