# Supplementary material for: Multinomial analysis of behavior: statistical methods
Source: Behav Ecol Sociobiol. 2017 Aug 25;71(9):138. doi: 10.1007/s00265-017-2363-8 (PMC5594044; doi:10.1007/s00265-017-2363-8)
Supplement: Supplementary file 1 — (PDF 1304 kb) [file 265_2017_2363_MOESM1_ESM.pdf]

# Multinomial Analysis of Behavior: Statistical Methods (Supplemental Figures and Tables)

Jeremy Koster[1] and Richard McElreath

[1]Corresponding author: jeremy.koster@uc.edu,

University of Cincinnati

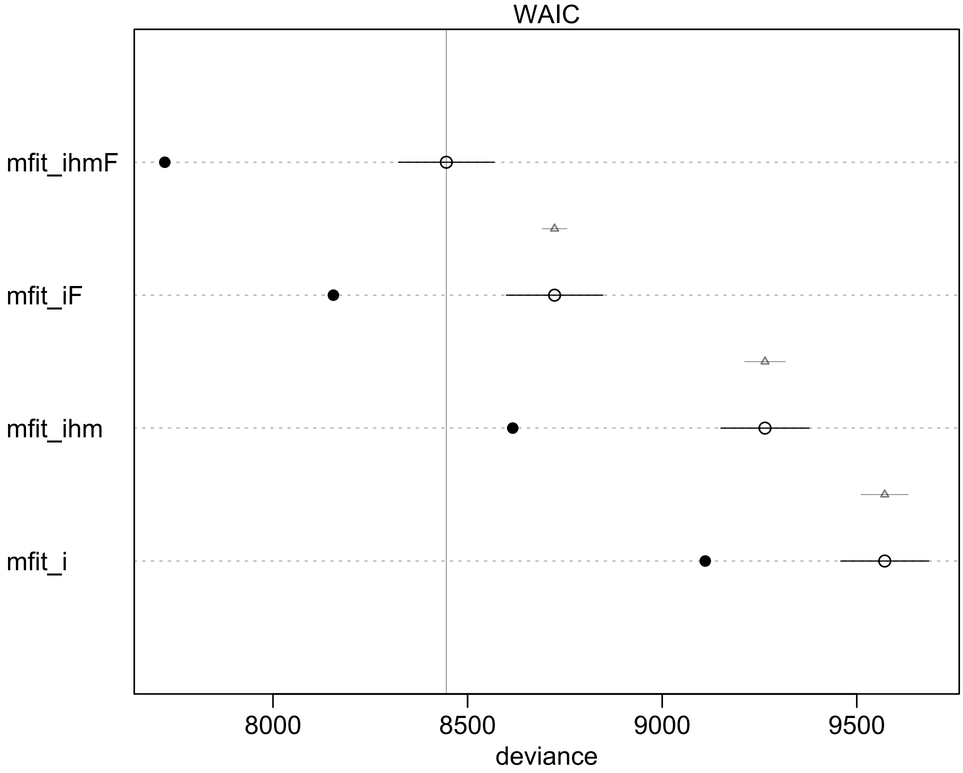


**Supplemental Fig. 1:** Visualization of WAIC values from Table 3. The open points are the WAIC for each candidate model. Dark lines through these points represent the standard deviation of the WAIC. The gray triangles above each WAIC is the difference between that model and top-ranking model, with the standard deviation of that difference depicted by gray segments. The filled points are the in-sample deviance of each model.

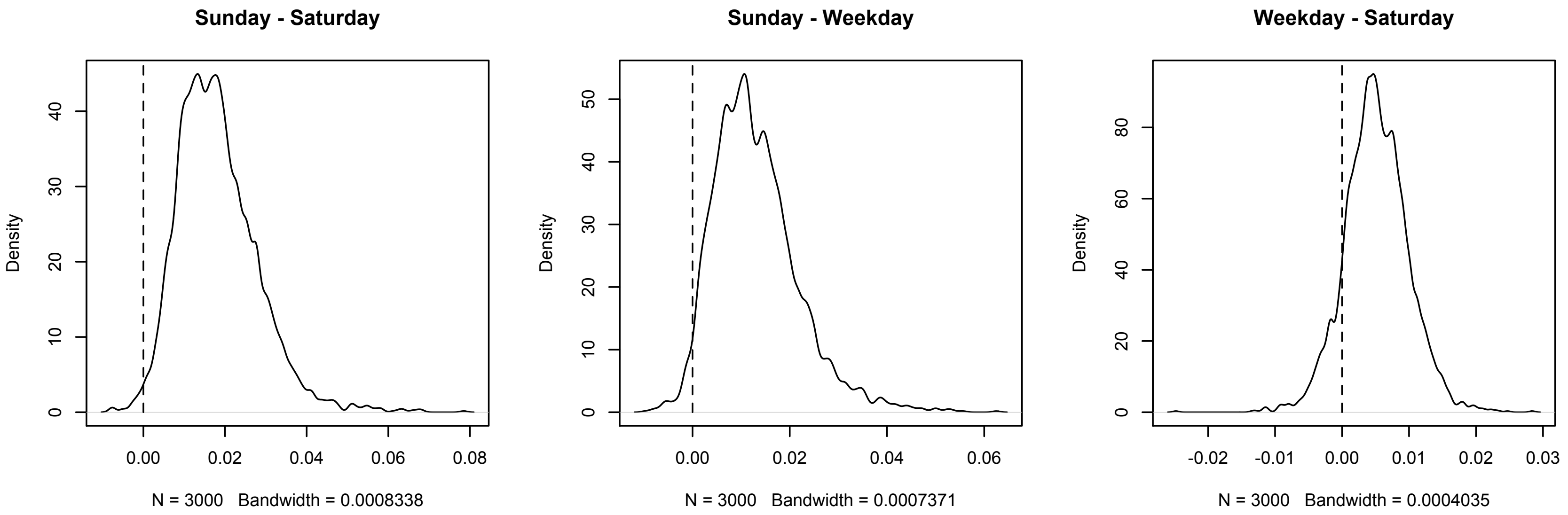


**Supplemental Fig. 2:** Distribution of predicted differences in the probability of other work on weekdays, Saturday, and Sunday, respectively. Predictions assume a time of 8:00 AM while all other covariates are held constant at the sample mean. Confidence intervals are 89% percentile intervals, as calculated from the posterior samples of Model *mfit_ iF*.

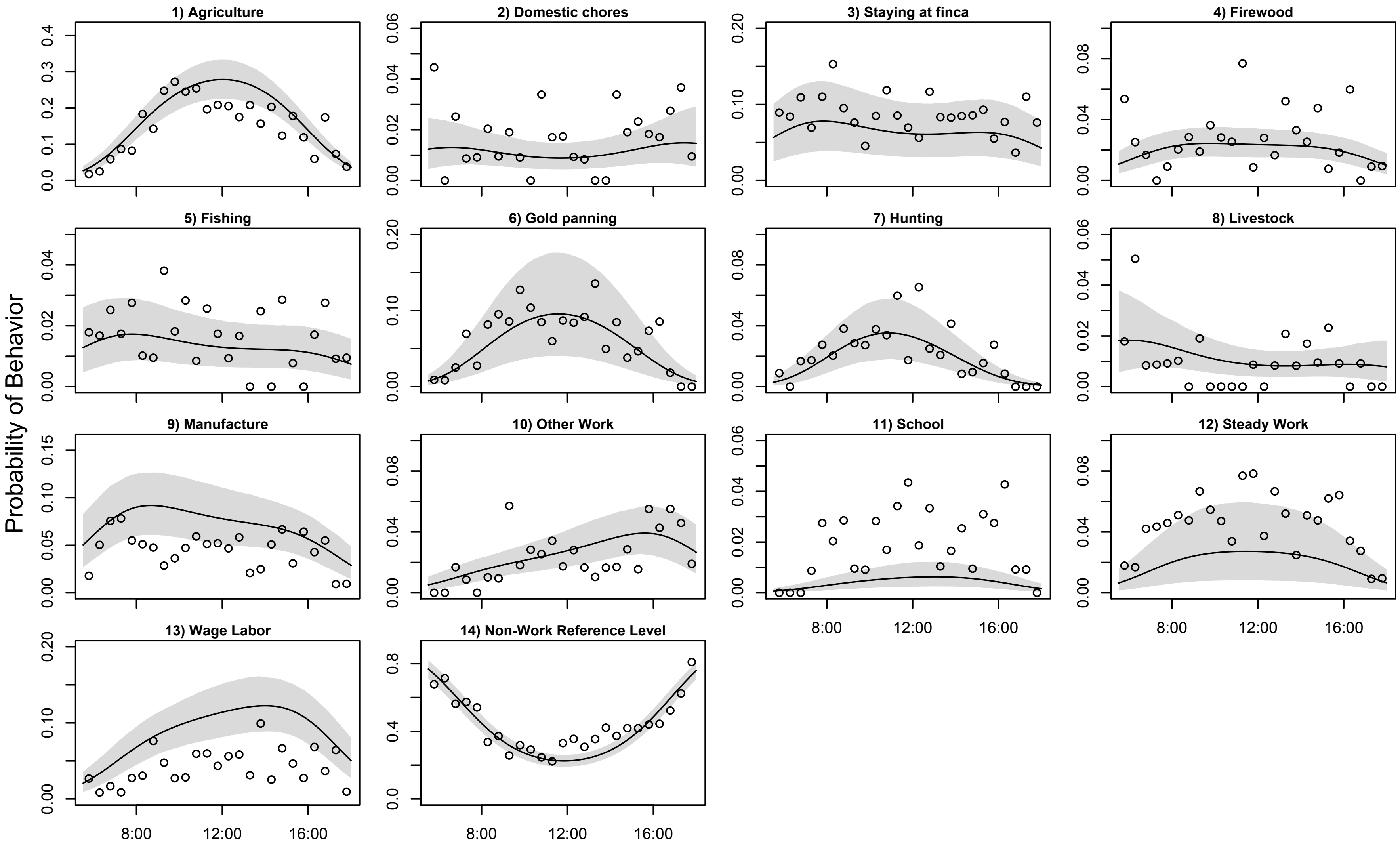


**Supplemental Fig. 3:** Model predictions of response behaviors as a function of time of day. All other covariates are held at the sample mean. Evident discrepancies between predictions and the empirical data, as in the prediction for School, stem from the values supplied for the other covariates. We held age constant at the sample mean, for example, whereas School is a common activity only for adolescent males. The shaded regions are the 89% percentile intervals, as calculated from the posterior samples of Model *mfit_ iF*.

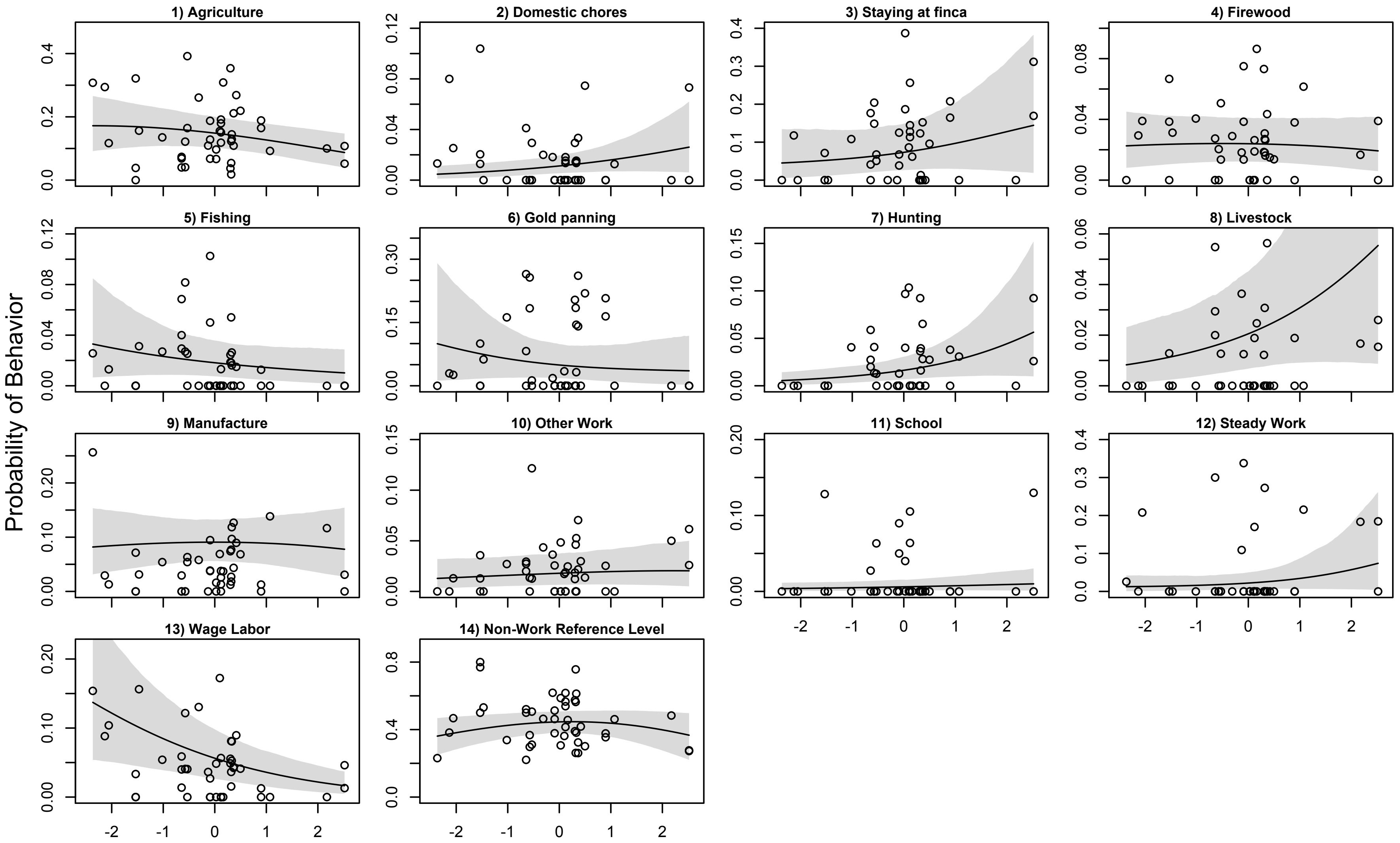


**Supplemental Fig. 4:** Model predictions of response behaviors as a function of household wealth, $z$-score standardized. The empirical data are binned in 30-minute intervals. Predictions assume a time of 8:00 AM while all other covariates are held constant at the sample mean. The shaded regions are the 89% percentile intervals, as calculated from the posterior samples of Model *mfit_ ihmF*.

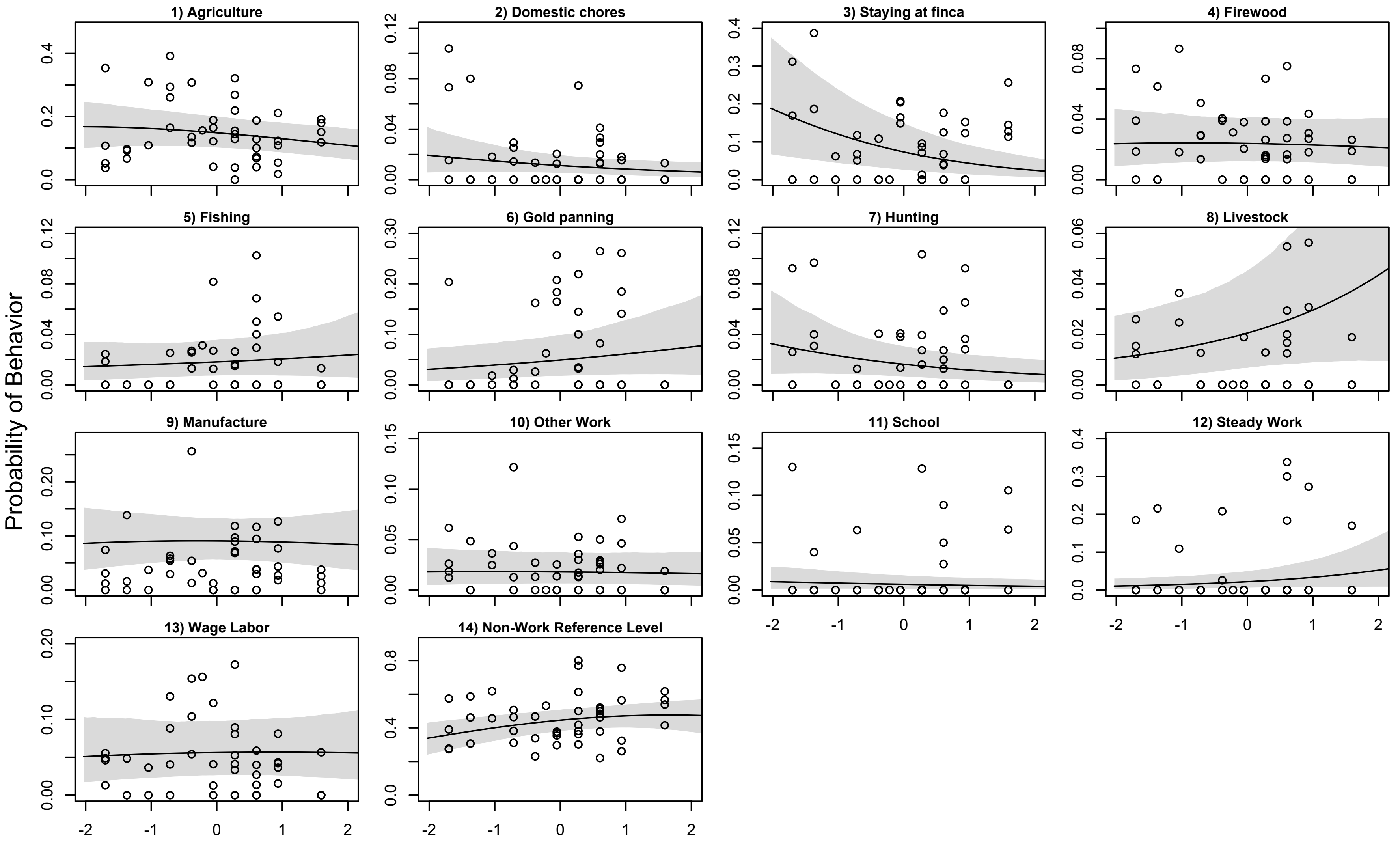


**Supplemental Fig. 5:** Model predictions of response behaviors as a function of household size, $z$-score standardized. Predictions assume a time of 8:00 AM while all other covariates are held constant at the sample mean. The shaded regions are the 89% percentile intervals, as calculated from the posterior samples of Model $mfit\_ihmF$.

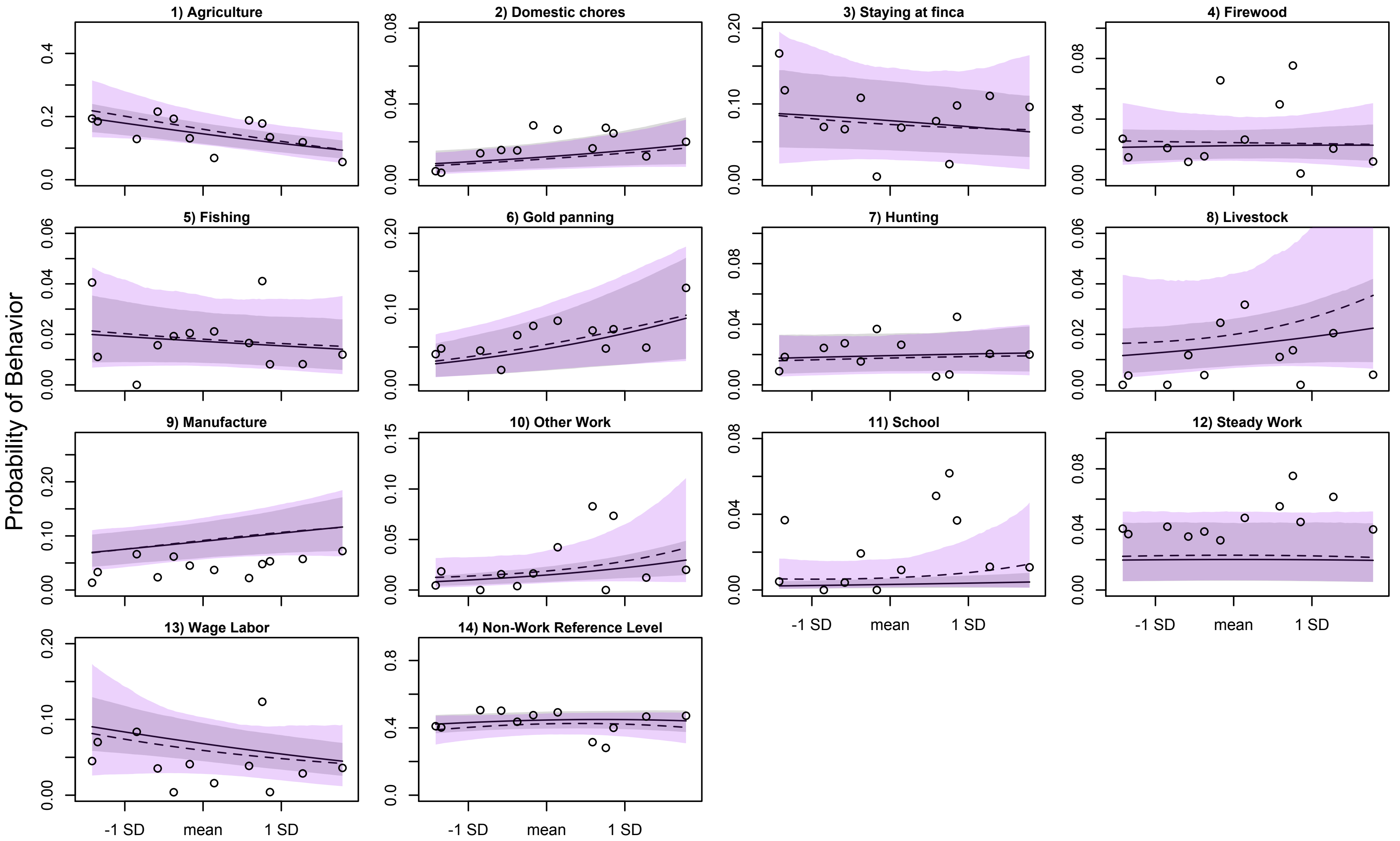


**Supplemental Fig. 6:** Model predictions of response behaviors as a function of monthly rainfall, *z*-score standardized. Predictions assume a time of 8:00 AM while all other covariates are held constant at the sample mean. We calculate predictions from two models, *mfit_ iF* and *mfit_ ihmF*, to show how the inclusion of the additional random effects generally increases uncertainty about model predictions. The solid line and gray scale represent predicted means and 89% percentile intervals from Model *mfit_ iF*. The dashed line and purple color depict the predicted means and 89% percentile intervals from Model *mfit_ ihmF*.

**Supplemental Table 1.** Posterior means (standard deviations in parentheses) of the intercepts in models *mfit_i* and *mfit_ihm*, respectively. Parameters in bold represent estimates whose 96% credible intervals do not include zero.

| | Agriculture | Domestic | Finca | Firewood | Fishing | Gold | Hunting | Livestock | Manu-facture | Other work | School | Steady work | Wage labor |
|---|---|---|---|---|---|---|---|---|---|---|---|---|---|
| ***mfit_i*** | | | | | | | | | | | | | |
| Intercept | **−1.15 (.13)** | **−3.51 (.27)** | **−2.14 (.31)** | **−2.82 (.15)** | **−3.44 (.25)** | **−3.02 (.40)** | **−3.12 (.27)** | **−3.77 (.25)** | **−2.44 (.21)** | **−2.98 (.20)** | **−4.48 (.41)** | **−4.20 (.50)** | **−2.50 (.22)** |
| ***mfit_ihm*** | | | | | | | | | | | | | |
| Intercept | **−1.06 (.24)** | **−3.42 (.34)** | **−2.19 (.50)** | **−2.60 (.36)** | **−3.19 (.48)** | **−2.71 (.51)** | **−3.13 (.34)** | **−3.29 (.69)** | **−2.40 (.26)** | **−2.46 (.65)** | **−3.13 (1.02)** | **−4.00 (.60)** | **−2.27 (.47)** |

**Supplemental Table 2.** Correlations of individual-level random effects across responses. The reported means are from the posterior samples (standard deviation in parentheses). Parameters in bold represent estimates whose 96% credible intervals do not include zero. The bottom half of the matrix depicts correlations from the model with random effects for individual, house, and month (*mfit_ihm*). This model lacks fixed effects. The top half of the matrix details correlations from the model with the full set of random effects and fixed effects (*mfit_ihmF*).

| | (1) | (2) | (3) | (4) | (5) | (6) | (7) | (8) | (9) | (10) | (11) | (12) | (13) |
|---|---|---|---|---|---|---|---|---|---|---|---|---|---|
| 1. Agriculture | | .04 (.23) | .33 (.20) | .03 (.25) | .05 (.24) | .03 (.21) | .06 (.22) | .04 (.24) | .12 (.25) | .12 (.24) | .05 (.24) | −.25 (.21) | −.02 (.24) |
| 2. Domestic | .02 (.21) | | .07 (.23) | .01 (.24) | .04 (.24) | .02 (.24) | .06 (.24) | .04 (.24) | −.04 (.25) | −.01 (.25) | .02 (.25) | −.02 (.23) | .00 (.24) |
| 3. Finca | .29 (.17) | .11 (.21) | | .03 (.25) | .05 (.24) | .27 (.20) | .32 (.23) | .02 (.23) | −.02 (.24) | .13 (.23) | .04 (.24) | −.31 (.18) | −.09 (.24) |
| 4. Firewood | .06 (.24) | .04 (.24) | .01 (.24) | | .02 (.25) | .06 (.24) | .02 (.24) | .02 (.25) | .03 (.25) | .00 (.25) | .02 (.25) | −.02 (.25) | −.02 (.25) |
| 5. Fishing | −.05 (.24) | .05 (.24) | .00 (.23) | .03 (.25) | | .06 (.25) | .07 (.24) | .00 (.26) | .02 (.25) | .03 (.25) | .02 (.25) | −.03 (.24) | .00 (.25) |
| 6. Gold | .01 (.18) | −.12 (.22) | .32 (.19) | −.02 (.24) | .02 (.24) | | .26 (.23) | .06 (.24) | .03 (.23) | .10 (.24) | −.02 (.25) | −.32 (.20) | −.06 (.24) |
| 7. Hunting | .07 (.21) | .00 (.23) | **.41 (.18)** | −.03 (.24) | .01 (.24) | .32 (.20) | | .03 (.25) | .02 (.25) | .11 (.25) | −.01 (.25) | −.12 (.21) | −.02 (.24) |
| 8. Livestock | .06 (.23) | .05 (.25) | .05 (.24) | .02 (.25) | −.01 (.25) | .08 (.24) | .04 (.25) | | .01 (.25) | .07 (.25) | .03 (.25) | −.02 (.24) | −.03 (.25) |
| 9. Manufacture | .35 (.18) | −.14 (.21) | .03 (.21) | −.02 (.24) | −.05 (.24) | .15 (.20) | .21 (.21) | .03 (.24) | | .01 (.24) | .00 (.25) | −.02 (.23) | .01 (.24) |
| 10. Other work | .37 (.18) | −.03 (.23) | .18 (.19) | −.04 (.24) | −.04 (.24) | .16 (.20) | .23 (.21) | .09 (.25) | .28 (.20) | | .05 (.25) | −.13 (.24) | −.03 (.25) |
| 11. School | −.23 (.19) | .21 (.20) | .06 (.21) | .06 (.24) | .12 (.24) | −.13 (.22) | −.10 (.21) | .03 (.24) | −.33 (.19) | −.22 (.20) | | −.07 (.24) | −.05 (.25) |
| 12. Steady | −.17 (.17) | −.05 (.22) | −.20 (.18) | −.07 (.24) | −.09 (.25) | −.19 (.20) | .04 (.20) | −.01 (.24) | .16 (.18) | .00 (.21) | −.23 (.20) | | .07 (.24) |
| 13. Wage | .25 (.18) | −.12 (.22) | .00 (.19) | −.08 (.24) | −.06 (.24) | .13 (.21) | .13 (.21) | −.03 (.24) | .30 (.19) | .23 (.21) | **−.43 (.19)** | .18 (.20) | |

**Supplemental Table 3.** Correlations of household-level random effects across responses. The reported means are from the posterior samples (standard deviation in parentheses). Parameters in bold represent estimates whose 96% credible intervals do not include zero. The bottom half of the matrix depicts correlations from the model with random effects for individual, house, and month (*mfit_ihm*). This model lacks fixed effects. The top half of the matrix details correlations from the model with the full set of random effects and fixed effects (*mfit_ihmF*).

| | (1) | (2) | (3) | (4) | (5) | (6) | (7) | (8) | (9) | (10) | (11) | (12) | (13) |
|---|---|---|---|---|---|---|---|---|---|---|---|---|---|
| 1. Agriculture | | −.03 (.24) | .15 (.24) | .03 (.24) | −.01 (.23) | −.06 (.23) | .00 (.24) | −.09 (.24) | .22 (.24) | .01 (.24) | .04 (.25) | .01 (.25) | .05 (.23) |
| 2. Domestic | −.02 (.24) | | −.02 (.24) | −.03 (.25) | .12 (.24) | −.02 (.23) | .05 (.24) | .09 (.24) | −.09 (.24) | .05 (.25) | −.02 (.24) | .02 (.25) | .10 (.24) |
| 3. Finca | .01 (.24) | .03 (.24) | | −.08 (.24) | −.01 (.22) | .00 (.22) | .17 (.25) | .01 (.24) | −.06 (.22) | .06 (.25) | .09 (.25) | −.01 (.25) | −.02 (.22) |
| 4. Firewood | .07 (.25) | −.01 (.23) | −.09 (.24) | | .06 (.24) | .02 (.23) | −.04 (.24) | .01 (.25) | .13 (.25) | −.02 (.25) | .02 (.25) | .04 (.25) | −.04 (.24) |
| 5. Fishing | −.04 (.24) | .06 (.23) | .02 (.22) | .03 (.24) | | .17 (.21) | .09 (.23) | .04 (.23) | .09 (.22) | −.01 (.24) | −.08 (.23) | .03 (.24) | .19 (.22) |
| 6. Gold | −.05 (.23) | −.02 (.22) | −.01 (.22) | .02 (.24) | .18 (.21) | | .18 (.22) | .14 (.22) | −.06 (.21) | .03 (.24) | −.12 (.24) | −.06 (.25) | .15 (.21) |
| 7. Hunting | −.09 (.24) | .12 (.24) | .19 (.25) | −.07 (.24) | .04 (.22) | .19 (.22) | | .11 (.23) | .04 (.22) | .13 (.25) | −.07 (.24) | −.01 (.25) | .18 (.23) |
| 8. Livestock | −.04 (.25) | .11 (.24) | .02 (.23) | .02 (.25) | .03 (.23) | .13 (.23) | .19 (.22) | | −.11 (.23) | .10 (.24) | .01 (.25) | .04 (.25) | −.01 (.23) |
| 9. Manufacture | .12 (.25) | −.03 (.24) | −.13 (.24) | .09 (.25) | .03 (.24) | −.02 (.24) | .13 (.23) | −.03 (.24) | | .02 (.24) | −.01 (.23) | .03 (.24) | .05 (.22) |
| 10. Other work | .02 (.24) | .08 (.24) | −.01 (.25) | .00 (.25) | −.04 (.25) | .05 (.24) | −.02 (.24) | .11 (.25) | .03 (.25) | | .02 (.25) | .01 (.24) | .02 (.24) |
| 11. School | −.01 (.25) | .01 (.24) | .12 (.25) | −.01 (.25) | .00 (.24) | −.11 (.25) | .05 (.24) | .00 (.24) | −.02 (.25) | .00 (.24) | | .02 (.25) | −.17 (.23) |
| 12. Steady | −.01 (.25) | .06 (.25) | .02 (.24) | .02 (.25) | .03 (.24) | −.03 (.25) | −.11 (.25) | .06 (.25) | .03 (.25) | .01 (.25) | .02 (.25) | | −.01 (.25) |
| 13. Wage | .04 (.25) | .10 (.24) | −.06 (.23) | −.04 (.25) | .15 (.23) | .21 (.22) | −.03 (.25) | .00 (.25) | .05 (.24) | .03 (.24) | −.10 (.25) | .01 (.24) | |

**Supplemental Table 4.** Correlations of month-level random effects across responses. The reported means are from the posterior samples (standard deviation in parentheses). Parameters in bold represent estimates whose 96% credible intervals do not include zero. The bottom half of the matrix depicts correlations from the model with random effects for individual, house, and month (*mfit_ihm*). This model lacks fixed effects. The top half of the matrix details correlations from the model with the full set of random effects and fixed effects (*mfit_ihmF*).

| | (1) | (2) | (3) | (4) | (5) | (6) | (7) | (8) | (9) | (10) | (11) | (12) | (13) |
|---|---|---|---|---|---|---|---|---|---|---|---|---|---|
| 1. Agriculture | | .02 (.26) | .07 (.21) | .07 (.22) | .12 (.23) | −.12 (.23) | .02 (.24) | −.01 (.23) | −.05 (.24) | .08 (.22) | .21 (.22) | .12 (.25) | −.02 (.21) |
| 2. Domestic | −.02 (.25) | | −.11 (.25) | .07 (.24) | .05 (.25) | .02 (.25) | .02 (.24) | .06 (.25) | .00 (.26) | .03 (.25) | .02 (.24) | .01 (.24) | .00 (.24) |
| 3. Finca | .09 (.20) | −.02 (.25) | | −.24 (.21) | −.02 (.22) | −.06 (.23) | −.01 (.24) | −.17 (.23) | −.01 (.23) | .04 (.20) | .16 (.21) | .05 (.23) | −.11 (.21) |
| 4. Firewood | .14 (.21) | .09 (.20) | −.19 (.21) | | .20 (.23) | .08 (.23) | −.10 (.24) | .19 (.22) | −.04 (.24) | .02 (.22) | .06 (.22) | .14 (.24) | .21 (.21) |
| 5. Fishing | .17 (.22) | .14 (.21) | .04 (.22) | .22 (.22) | | .08 (.24) | −.07 (.25) | .14 (.24) | −.08 (.24) | .08 (.23) | .17 (.23) | .12 (.24) | .03 (.23) |
| 6. Gold | −.20 (.21) | .17 (.22) | −.02 (.22) | .11 (.22) | .06 (.23) | | −.04 (.24) | .06 (.24) | .02 (.25) | .10 (.23) | .03 (.24) | .00 (.24) | −.01 (.24) |
| 7. Hunting | −.03 (.25) | −.20 (.21) | −.04 (.24) | −.05 (.24) | −.03 (.25) | .02 (.24) | | −.01 (.25) | .03 (.25) | .04 (.24) | −.01 (.24) | −.08 (.25) | −.09 (.25) |
| 8. Livestock | −.02 (.22) | −.03 (.25) | −.10 (.23) | .23 (.22) | .15 (.24) | .12 (.23) | .03 (.24) | | −.07 (.25) | .13 (.22) | .05 (.24) | .05 (.24) | .05 (.23) |
| 9. Manufacture | −.08 (.24) | −.02 (.22) | −.03 (.24) | .00 (.24) | −.07 (.24) | .12 (.24) | .02 (.25) | −.01 (.25) | | −.12 (.25) | −.02 (.25) | −.02 (.25) | −.01 (.24) |
| 10. Other work | .03 (.21) | −.08 (.24) | .08 (.21) | .05 (.21) | .09 (.23) | .23 (.21) | .08 (.25) | .20 (.23) | −.04 (.24) | | .23 (.21) | .02 (.24) | −.10 (.21) |
| 11. School | .18 (.22) | .03 (.21) | .24 (.21) | .11 (.21) | .20 (.23) | .19 (.23) | .02 (.25) | .16 (.24) | .05 (.24) | .32 (.20) | | .14 (.24) | .03 (.22) |
| 12. Steady | .14 (.23) | .18 (.22) | .07 (.23) | .20 (.24) | .18 (.24) | .04 (.24) | −.05 (.25) | .10 (.24) | −.01 (.25) | .05 (.24) | .18 (.25) | | .10 (.23) |
| 13. Wage | .08 (.20) | .14 (.23) | −.05 (.21) | .25 (.21) | .08 (.22) | −.02 (.22) | −.06 (.25) | .07 (.23) | .00 (.24) | −.07 (.21) | .08 (.22) | .14 (.24) | |
