# Supplementary material for: Multinomial analysis of behavior: statistical methods
Source: Behav Ecol Sociobiol. 2017 Aug 25;71(9):138. doi: 10.1007/s00265-017-2363-8 (PMC5594044; doi:10.1007/s00265-017-2363-8)
Supplement: Supplementary file 2 — (DOCX 65 kb) [file 265_2017_2363_MOESM2_ESM.docx]

Multinomial Analysis of Behavior: Statistical Methods

(Supplemental File 1: Statistical Notation)

Jeremy Koster^1^ and Richard McElreath

^1^ Corresponding author: jeremy.koster@uc.edu, University of Cincinnati

This notation is for models that include varying intercepts for household and month in addition to varying intercepts for individuals (*v*). As in the text, this notation makes the simplifying assumption that there are only three response categories.

$$\text{log}\left( \frac{\pi_{\text{1}it}}{\pi_{\text{3}it}} \right)=\beta_{\text{1}it}+v_{\text{1}i}+{v\_house}_{1i}+{v\_month}_{1i}$$

$$\text{log}\left( \frac{\pi_{\text{2}it}}{\pi_{\text{3}it}} \right)=\beta_{\text{2}it}+v_{\text{2}i}+{v\_house}_{2i}+{v\_month}_{2i}$$

$$\left[ \begin{matrix} v_{\text{1}i} \\ v_{\text{2}\text{i}} \end{matrix} \right]\sim\text{Normal(0,}\Omega_{v}):\Omega_{v}=\left[ \begin{matrix} \sigma_{v\text{1}}^{2} & \\ \sigma_{v\text{1,2}} & \sigma_{v\text{2}}^{2} \end{matrix} \right]$$

$$\left[ \begin{matrix} {v\_house}_{\text{1}i} \\ {v\_house}_{\text{2}\text{i}} \end{matrix} \right]\sim\text{Normal(0,}\Omega_{v}):\Omega_{v}=\left[ \begin{matrix} \sigma_{v\_house\text{1}}^{2} & \\ \sigma_{v\_house\text{1,2}} & \sigma_{v\_house\text{2}}^{2} \end{matrix} \right]$$

$$\left[ \begin{matrix} {v\_month}_{\text{1}i} \\ {v\_month}_{\text{2}\text{i}} \end{matrix} \right]\sim\text{Normal(0,}\Omega_{v}):\Omega_{v}=\left[ \begin{matrix} \sigma_{v\_month\text{1}}^{2} & \\ \sigma_{v\_month\text{1,2}} & \sigma_{v\_month\text{2}}^{2} \end{matrix} \right]$$

$$\pi_{\text{1}}+\pi_{\text{2}}+\pi_{\text{3}}=1$$
